# Safety Monitoring of Colistin Therapy in Critically Ill Neonates With Late‐Onset Sepsis: A Retrospective Observational Study

**DOI:** 10.1002/prp2.70178

**Published:** 2025-09-30

**Authors:** Baran Cengiz Arcagok, Akan Yaman, Turkay Rzayev, Nazli Jalalzada, Ibrahim Kandemir, Asli Memisoglu, Hulya Selva Bilgen

**Affiliations:** ^1^ Department of Pediatrics, Division of Neonatology, School of Medicine Acibadem University Istanbul Turkey; ^2^ Nisantasi University Istanbul Turkey; ^3^ Department of Pediatrics, Division of Neonatology, School of Medicine Marmara University Istanbul Turkey; ^4^ Department of Pediatrics Istanbul Health and Technology University Istanbul Turkey

**Keywords:** colistin, hypomagnesemia, late‐onset sepsis, multidrug‐resistant microorganisms, neonatal intensive care unit, neonate

## Abstract

This study aimed to evaluate the safety of colistin therapy by monitoring renal function and electrolyte levels in critically ill neonates with late‐onset sepsis (LOS) hospitalized in the neonatal intensive care unit (NICU) between 2015 and 2021. This retrospective case–control study included 58 critically ill neonates treated with colistin for late‐onset sepsis and 22 control neonates with late‐onset sepsis who did not receive colistin. Data were analyzed to compare patient outcomes, microbiological profiles, and side effects of treatment. Statistical analyses were performed using repeated‐measures ANOVA and Bayesian calculations to evaluate serum creatinine levels and biochemical parameters over time. Serum creatinine levels showed similar alterations within the first 7 days of colistin treatment with moderate evidence. However, serum magnesium and sodium levels were lower on the 7th day in the colistin‐treated group compared with the control group. Colistin therapy in critically ill neonates with late‐onset sepsis appears to be a viable treatment option with an acceptable short‐term safety profile. These findings emphasize the importance of routine monitoring of renal function and electrolyte levels during colistin use in neonatal intensive care to minimize potential complications.

## 1 Introduction

Multidrug‐resistant (MDR) microorganisms cause infections with high mortality and morbidity rates in hospital settings, particularly in intensive care units [1, 2]. In recent years, the global incidence of infections due to MDR microorganisms, especially bacteria such as *Pseudomonas aeruginosa* , *Acinetobacter baumannii* , and *Klebsiella pneumoniae* , has been increasing [3]. Due to the limited availability of drugs to treat these pathogens, the use of colistin, one of the last‐resort antibiotics, has been rising worldwide [4]. With the increased use of colistin, known for its effectiveness against MDR aerobic Gram‐negative bacteria, its side effects have also come under discussion. There is limited information in the literature regarding the safety of colistin treatment in newborns. Although the few studies that examine the efficacy and side effects of colistin therapy in newborns provide valuable insights [4, 5], the presence of comorbid conditions and the concurrent use of other antibiotics in newborns complicate research efforts and the interpretation of studies' results. Therefore, it is essential to continue researching the safety of colistin treatment in newborns and to keep sharing experiences on this topic. The primary aim of this study was to evaluate the safety of colistin therapy by monitoring renal function and electrolyte levels in critically ill newborns with late‐onset sepsis (LOS) who were hospitalized in the Neonatal Intensive Care Unit (NICU) between 2015 and 2021.

## 2 Materials and Methods

### 2.1 Patient Population

This study was conducted in the tertiary neonatal intensive care unit of a university hospital, providing a specialized setting for the care and study of critically ill neonates. Patients diagnosed with LOS and treated with colistin (*n*: 58) while hospitalized in the NICU between 2015 and 2021 were included in the hospital's electronic operating system. Colistin therapy was initiated based on blood culture results indicating carbapenem‐resistant microbial growth in neonates with elevated acute‐phase reactants. The decision to initiate colistin was made in consultation with pediatric infectious disease specialists. In all cases, colistin treatment was indicated following the confirmation of carbapenem resistance and the presence of clinical signs consistent with severe infection. In a small number of critically ill neonates with negative or inconclusive cultures, colistin was initiated empirically based on clinical deterioration and strong suspicion of MDR infection, in consultation with pediatric infectious disease specialists. LOS was diagnosed in neonates older than 72 h of life based on a combination of clinical and laboratory findings. Clinical signs included tachycardia (heart rate > 160/min) or bradycardia (heart rate < 60/min), temperature instability, feeding intolerance, respiratory distress, increased need for oxygen, recurrent apnoea, hypotonia, and lethargy. Laboratory parameters included elevated CRP (> 10 mg/L), leukopenia or leukocytosis (WBC < 5000/mm^3^ or > 25 000/mm^3^), immature/total neutrophil count > 0.2, thrombocytopenia (< 150 000/mm^3^), or culture‐proven bacterial growth.

The control group, consisting of neonates with LOS who were not treated with colistin, was selected by matching with the study group. These neonates were hospitalized during the same period, treated according to the same care standards, and followed up with the same nursing care and medical interventions. A total of 22 neonates were included in the control group, which was randomized using a block randomization method. The relatively smaller control group size reflects the limited number of neonates with LOS who did not require colistin and simultaneously fulfilled the strict inclusion criteria with sufficiently complete data. Although additional LOS cases were present during the study period, their inclusion would have introduced heterogeneity or relied on incomplete records. Therefore, only 22 neonates were eligible for the control group.

The inclusion criteria for the study were defined separately for the colistin‐treated group and the control group. For the colistin‐treated group, patients were included if they were hospitalized in the neonatal intensive care unit (NICU) between 2015 and 2021, diagnosed with late‐onset sepsis (LOS) based on clinical signs appearing after the third postnatal day, and received colistin therapy during their hospitalization. For the control group, patients were included if they were hospitalized in the NICU during the same period (2015–2021), diagnosed with late‐onset sepsis (LOS) with clinical signs occurring after the third postnatal day, but did not receive colistin therapy during their hospitalization.

Neonates were excluded from the study if they had severe congenital anomalies or chromosomal disorders, such as trisomy 13 or trisomy 18, which could independently influence clinical outcomes. Patients with significant pre‐existing renal, hepatic, or cardiac dysfunction (including hemodynamically significant patent ductus arteriosus) unrelated to the sepsis episode were also excluded. Furthermore, infants with incomplete or missing critical clinical data, including essential laboratory results or sepsis‐related parameters, were not included in the analysis to ensure the reliability of the study findings.

### 2.2 Study Design and Data Collection

This study was designed as a retrospective case–control study. Data were collected by reviewing patients' electronic medical records, patient files, and nursing records. Information on the patients' gender, delivery type, gestational age, birth weight, chronological age at the time of hospitalization, length of hospital stay, and primary diagnoses was recorded. Additionally, the presence of maternal conditions such as chorioamnionitis, gestational diabetes, preeclampsia, premature rupture of membranes, and the administration of antenatal steroids was investigated.

The presence of an umbilical catheter, early‐onset sepsis (within the first three days), LOS, clinical sepsis, culture‐proven sepsis, day of hospitalization, day of colistin treatment, infection site, type of microbial agent, total cumulative dose of treatment, and eradication time were recorded. Colistin was administered intravenously as colistimethate sodium. The standard regimen consisted of 2.5 mg/kg every 12 h, adjusted according to renal function. Treatment duration ranged between 7 and 21 days (median: 12 days). The presence of comorbid gastroenterological, metabolic, endocrinological, neurological, and respiratory conditions during sepsis, as well as the need for mechanical ventilation, metabolic acidosis, low platelet count, anemia, and hemodynamic instability, was also investigated.

The baseline levels of sodium (Na), potassium (K), calcium (Ca), magnesium (Mg), blood urea nitrogen (BUN), and creatinine before the initiation of colistin treatment were recorded, along with their levels on the first, third, and seventh days of treatment. For patients receiving prolonged treatment, weekly levels were recorded after the seventh day.

### 2.3 Ethics Committee Approval

The study was conducted in accordance with the principles of the 2013 Declaration of Helsinki. Ethics committee approval was obtained (Dean of Faculty Clinical Research Ethics Committee, Date: 02.07.2021; Number: 09.2021.885).

Informed consent was obtained from the parents of the patients involved in this study.

### 2.4 Statistical Analysis

We estimated that 74 patients (55 case, 19 control) would be needed to detect (with a probability greater than 0.9) an effect size of *d* = 0.8 in a student's *t* test model with a type I error rate of less than α = 0.05.

We presented data as *n* (%), mean ± standard deviation (SD), and median (interquartile range [IQR]), depending on the distribution, which was assessed using the Kolmogorov–Smirnov test, including skewness, kurtosis, and Q‐Q plots. We used student's *t* test or Mann–Whitney *U* test to compare two independent groups after testing normality. In case of normally distributed non‐homogeneous groups (homogeneity tested with Levene's test), we used Welch *t*‐test. Also, we used the repeated‐measures ANOVA (RM‐ANOVA) test to assess changes across multiple measurements and applied the Greenhouse–Geisser correction if the sphericity assumption was not met. Missing data (< 20%) primarily involved electrolyte levels at later time points, most often due to sampling difficulties or early discharge. These were considered missing completely at random (MCAR). Multiple imputation was applied to prevent data loss in RM‐ANOVA models. Another calculation we used was Bayesian methods. We formulated the H1 (significant difference) and H0 (independence) hypotheses for Bayesian RM‐ANOVA and reported the Bayes factors. Additionally, we presented estimated marginal means tables with 95% confidence intervals (95% CI). All calculations were performed using the SPSS 29 package and JAMOVI 2.3.18 package (with the jsq extension).

## 3 Results

A total of 80 patients (58 cases, 22 controls) were included in the study, 46.3% (*n* = 37) of whom were female. The descriptive characteristics and serum creatinine levels are summarized in Table 1. The median postnatal day at sepsis onset was 22 days (IQR: 7–44).

**TABLE 1:** Comparison of descriptive features and creatinine levels of the patients.

	Treatment included Colistin (*n* = 58)	Treatment without Colistin (*n* = 22)	*p*
Gender (Female)	48.3% (*n* = 28)	40.9% (*n* = 9)	*0.555* ^C^
Gestational week	31.9 ± 5.0	31.4 ± 5.9	0.695^S^
Birth weight	1500 (910–2530)	1330 (970–2580)	0.991^M^
Total hospitalization time	81.0 ± 54.5	96.7 ± 66.9	0.283^S^
Postnatal day of sepsis onset	20.0 (5.0–44.3)	24.5 (12‐42.5)	0.176^M^
Basal creatinine	0.39 ± 0.22	0.37 ± 0.15	0.753^S^
1st day‐creatinine	0.35 ± 0.19	0.38 ± 0.15	0.586^S^
3rd day‐creatinine	0.33 ± 0.18	0.36 ± 0.15	0.609^S^
7th day‐creatinine	0.32 ± 0.16	0.29 ± 0.09	0.511^S^
14th day‐creatinine	0.30 ± 0.14	0.26 ± 0.11	0.577^S^
Mortality (due to sepsis)	6.9% (*n* = 4)	4.5% (*n* = 1)	1^F^

*Note:* Data is presented as mean ± SD, median (IQR), or % (*n*).

Abbreviations: C, Chi‐square test; F, Fisher's exact test; M, Mann–Whitney *U* test; S, Student's *t* test; W, Welch's *t* test.

Microbiological cultures showed no growth in 35.0% (*n* = 28) of cases. Among the identified pathogens, *Acinetobacter baumannii* was the most common (23.8%, *n* = 19), followed by gram‐negative bacteria (21.3%, *n* = 17; including *Klebsiella pneumoniae, Pseudomonas aeruginosa, Stenotrophomonas maltophilia, Enterobacter* spp., and *Ralstonia pickettii* ), gram‐positive bacteria (18.8%, *n* = 15; including *Staphylococcus* spp., *Streptococcus* spp., *Microbacterium flavescens laeveniformis*, and *Enterococcus* spp.), and *Candida albicans* in one case.

The relationships between serum creatinine levels (at baseline, day 1, day 3, and day 7) and various clinical factors were assessed. A correlation was observed between anemia during sepsis and 7th‐day creatinine levels; however, this was not statistically significant in Welch's *t*‐test (*p* = 0.182). No meaningful associations were found between serum creatinine levels and the following variables: LGA/SGA/AGA status, colistin treatment, delivery type, history of umbilical venous catheterization (UVC), need for mechanical ventilation during sepsis, perfusion impairment, metabolic acidosis, thrombocytopenia, anemia (on days other than day 7), or necrotising enterocolitis (NEC) history (Table 2).

**TABLE 2:** Correlations of serum creatinine follow‐up with the clinical features.

	Serum creatinine							
	Basal		1st day		3rd day		7th day	
	*r*	BF_10_	*r*	BF_10_	*r*	BF_10_	*r*	BF_10_
Colistin treatment	−0.023	0.152	−0.080	0.253	−0.088	0.272	−0.021	0.174
Sex	0.117	0.465	0.175	0.981	0.090	0.280	0.030	0.179
SGA/AGA/LGA	0.034	0.160	−0.001	0.177	0.022	0.172	−0.059	0.210
Delivery	0.006	0.146	0.045	0.198	−0.046	0.190	−0.051	0.199
UVC	0.013	0.148	0.103	0.318	0.089	0.276	0.111	0.359
MV need during sepsis	−0.087	0.279	−0.074	0.240	0.030	0.177	−0.179	1.224
Perfusion impairment during sepsis	0.081	0.254	0.099	0.305	0.077	0.242	0.006	0.170
Metabolic acidosis during sepsis	0.045	0.173	0.041	0.194	0.023	0.172	−0.016	0.172
Thrombocytopenia during sepsis	0.019	0.150	0.059	0.215	−0.044	0.188	−0.024	0.176
Anemia during sepsis attack	−0.030	0.158	−0.032	0.187	−0.039	0.183	−0.205	2.243

*Note:* Bayesian Kendall's tau test.

**FIGURE 1:**
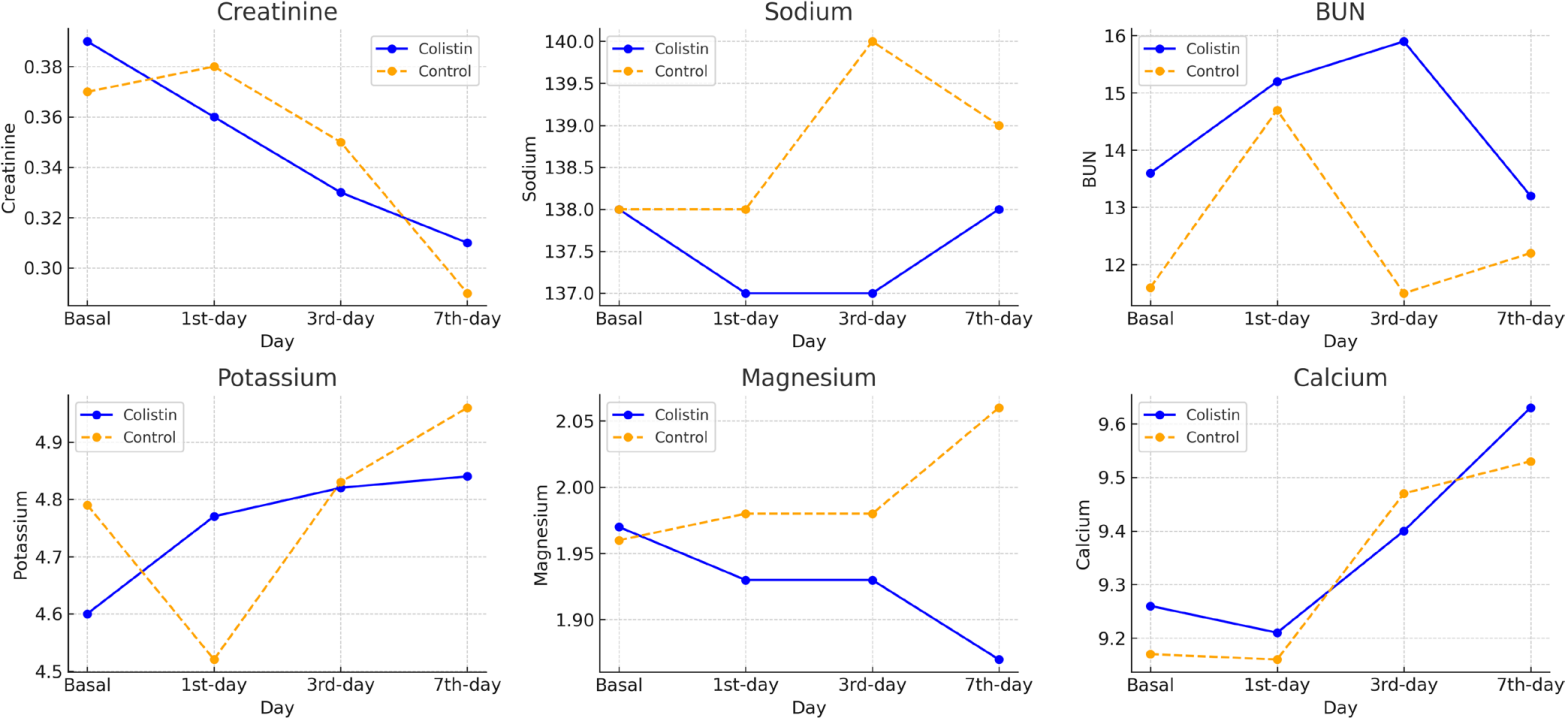
Alterations in biochemical parameters in neonates with and without Colistin Treatment.

Bayesian RM‐ANOVA revealed moderate evidence for independence between colistin use and creatinine levels (BF_10_ = 0.137). Similarly, when adjusting for serum creatinine changes, colistin treatment still showed moderate evidence for independence (BF_10_ = 0.270). Conversely, when assuming no effect of colistin treatment, creatinine levels changed significantly over time in both groups (BF_10_ = 45.45). Frequentist RM‐ANOVA indicated that creatinine levels on day 7 were significantly lower compared to baseline (*p* = 0.005) and day 1 (*p* = 0.012, Tukey pairwise test). Estimated marginal means are shown in Table 3 and visualized in Figure 1.

**TABLE 3:** Estimated marginal means table of serum creatinine, serum sodium, BUN, serum potassium, serum magnesium, and serum calcium regarding the initiation of and the day of onset of colistin treatment (with 95% confidence intervals).

Colistin	Days	Expected Means					
		Creatinine_(mg/dL)_	Sodium_(mEq/L)_	BUN_(mg/dL)_	Potassium_(mEq/L)_	Magnesium_(mEq/L)_	Calcium_(mg/dL)_
No	Basal	0.37 (0.28–0.46)	138 (136–139)	11.6 (8.6–14.5)	4.79 (4.47–5.11)	1.96 (1.83–2.09)	9.17 (8.88–9.47)
	1st‐day	0.38 (0.31–0.44)	138 (136–140)	14.7 (11.3–18.0)	4.52 (4.21–4.83)	1.98 (1.89–2.08)	9.16 (8.83–9.49)
	3rd‐day	0.35 (0.29–0.42)	140 (138–141)	11.5 (7.4–15.5)	4.83 (4.56–5.11)	1.98 (1.89–2.07)	9.47 (9.21–9.73)
	7th‐day	0.29 (0.24–0.34)	139 (138–140)	12.2 (9.6–14.9)	4.96 (4.64–5.28)	2.06 (1.95–2.17)	9.53 (9.24–9.81)
Yes	Basal	0.39 (0.33–0.44)	138 (137–139)	13.6 (11.8–15.4)	4.60 (4.41–4.80)	1.97 (1.89–2.05)	9.26 (9.08–9.44)
	1st‐day	0.36 (0.32–0.39)	137 (136–138)	15.2 (13.1–17.3)	4.77 (4.58–4.96)	1.93 (1.87–1.99)	9.21 (9.01–9.41)
	3rd‐day	0.33 (0.29–0.37)	137 (136–138)	15.9 (13.5–18.4)	4.82 (4.65–4.99)	1.93 (1.87–1.98)	9.40 (9.24–9.56)
	7th‐day	0.31 (0.28–0.35)	138 (137–138)	13.2 (11.6–14.8)	4.84 (4.64–5.03)	1.87 (1.80–1.94)	9.63 (9.45–9.80)

Bayesian RM‐ANOVA gave moderate evidence for correlation between colistin use and serum sodium levels (BF_10_ = 8.48). After adjusting for serum sodium alterations, colistin treatment showed anecdotal evidence for colistin effect (BF_10_ = 1.6). However, when assuming no effect of colistin treatment, serum sodium levels were similar over time in both groups with strong evidence (BF_10_ = 0.06). In frequentist ANOVA calculations, serum sodium levels were lower in the colistin group (*p* = 0.034) in pairwise comparisons (Tukey pairwise comparison test). Estimated marginal means are shown in Table 3 and visualized in Figure 1.

Regarding BUN levels, Bayesian analysis indicated no clear evidence of a colistin effect (BF_10_ = 1.07). Adjusted models for colistin (assuming BUN alteration has a null effect) provided anecdotal evidence for independence (BF_10_ = 0.55) for colistin effect, and BUN levels (assuming colistin has a null effect) remained stable over time in both groups with moderate evidence (BF_10_ = 0.289). Estimated marginal means are shown in Table 3 and visualized in Figure 1.

Serum potassium alterations were similar between the colistin group and the control, with moderate evidence (BF10: 0.14) in pairwise comparisons. The adjusted effect of colistin (assuming serum potassium alterations have a null effect) gave moderate evidence for independence (BF_10_: 0.189), and serum potassium levels remained stable (assuming colistin has a null effect) with moderate evidence (BF_10_ = 0.173).

For serum magnesium levels, colistin showed anecdotal evidence of an effect (BF_10_ = 1.58), but overall evidence supported independence with barely anecdotal evidence (BF_10_ = 0.822). Magnesium levels remained stable (assuming colistin has a null effect) in both groups with strong evidence (BF_10_ = 0.027). In frequentist RM‐ANOVA analysis, magnesium levels were remarkably lower in the colistin group, but without statistical significance (*p* = 0.064).

Regarding serum calcium, there was moderate evidence suggesting no effect of colistin in Bayesian RM‐ANOVA and in a model where serum calcium alteration was assumed to have a null effect (BF_10_ = 0.154 and 0.229, respectively). However, calcium levels significantly increased over time in both groups with extreme evidence (BF_10_ = 412.0). Frequentist RM‐ANOVA analysis showed that 7th‐day calcium levels were higher than baseline (*p* = 0.002) and day 1 (*p* = 0.007).

Overall, the findings provide compelling evidence that colistin treatment in critically ill neonates with late‐onset sepsis is not associated with clinically significant nephrotoxicity or major electrolyte disturbances, supporting its continued use with appropriate monitoring.

## 4 Discussion

Previous studies have highlighted colistin's critical role in treating MDR infections, especially those caused by *Pseudomonas aeruginosa* and *Acinetobacter baumannii* , pathogens frequently encountered in NICUs [5, 11]. Colistin's bactericidal mechanism underscores its importance as a last‐resort antibiotic in severe neonatal infections. Our findings suggest that colistin remains an important treatment option for managing life‐threatening infections caused by MDR pathogens in neonates, though further research is needed to assess its long‐term safety and efficacy [5, 10, 11, 13, 14, 15].

While colistin remains a crucial option in the management of multidrug‐resistant infections in neonates, concerns about its nephrotoxic and neurotoxic potential—particularly in patients with immature renal function and underdeveloped blood–brain barriers—persist. Previous pediatric studies have reported nephrotoxicity rates of up to 22% and neurotoxicity in approximately 4% of patients receiving colistin for more than 72 h [6, 7]. However, in our study, Bayesian repeated‐measures ANOVA showed moderate evidence supporting the independence between colistin use and changes in serum creatinine levels. Frequentist RM‐ANOVA also indicated that serum creatinine levels significantly decreased over time in both groups, particularly by day 7 compared to baseline (*p* = 0.005), with no distinct nephrotoxic pattern in the colistin‐treated group. This trend may be explained by improved renal perfusion during recovery from sepsis, rather than a renal impact of colistin itself. These findings suggest that, under close monitoring, colistin may not significantly impair renal function in neonates, contrasting with earlier studies in older pediatric populations.

Furthermore, our analysis identified a statistically significant difference in serum sodium levels in the colistin group (*p* = 0.034) and a trend toward lower magnesium levels (*p* = 0.064). These findings are consistent with reports highlighting the risk of electrolyte disturbances, particularly hypomagnesemia, in very low birth weight infants receiving colistin [8, 9, 12]. Electrolyte imbalances in this population can lead to serious clinical consequences, including cardiovascular instability and neurologic symptoms, underscoring the importance of close biochemical surveillance during treatment. Despite no clear association with potassium, calcium, or BUN alterations, the observed shifts in sodium and magnesium reinforce the need for individualized electrolyte management protocols when using colistin in neonatal intensive care.

Hypocalcemia is a common metabolic disturbance in neonates with late‐onset sepsis and may gradually improve during the recovery phase [17]. In the present study, we observed hypocalcemia in a subset of neonates receiving colistin therapy. Although electrolyte disturbances are not widely recognized among the direct side effects of colistin, its known nephrotoxic potential may indirectly disrupt calcium homeostasis. Additionally, hypocalcemia in septic neonates has been associated with the systemic inflammatory response, altered parathyroid hormone secretion, and inadequate calcium intake (20). While our Bayesian RM‐ANOVA model showed no direct effect of colistin on serum calcium levels (BF_10_ = 0.154 and 0.229), calcium levels significantly increased over time in both groups (BF_10_ = 412.0; *p* = 0.002 vs. baseline, *p* = 0.007 vs. day 1), indicating spontaneous recovery during clinical stabilization. These findings emphasize the importance of regular electrolyte monitoring in critically ill neonates, particularly those receiving potentially nephrotoxic treatments such as colistin.

The risk of neurotoxicity associated with colistin, including potential peripheral neuropathy, neuromuscular blockade, and other neurodevelopmental delays, has been documented in previous studies [6, 12, 16]. Although no overt signs of neurotoxicity were observed in our cohort during hospitalization, this study did not include specific neurologic evaluations such as EEG, neuroimaging, or ABR testing. Furthermore, the lack of long‐term follow‐up limits conclusions about delayed neurodevelopmental effects. These limitations underscore the importance of cautious use of colistin and the need for prospective studies evaluating its potential neurotoxic impact in neonates.

Our study stands out by evaluating renal and electrolyte effects of colistin therapy in neonates using both Bayesian and frequentist statistical methods, offering a more nuanced and robust analysis than many previous reports. Unlike studies conducted in broader pediatric populations, our findings provide focused insight into the hemodynamic and biochemical changes specifically in preterm and term neonates during colistin therapy. The lack of significant nephrotoxicity in our cohort may reflect differences in dosing strategies, improved supportive care, or earlier intervention in the neonatal intensive care setting. Moreover, the stability of creatinine levels despite ongoing colistin exposure supports the notion that, with vigilant fluid and perfusion management, renal safety can be maintained.

Nevertheless, this study has several limitations. The retrospective design and moderate rate of missing data (< 20% total) may reduce the statistical power and introduce bias. Additionally, the absence of urine output data and more sensitive renal biomarkers, such as NGAL or cystatin C, limits our ability to fully assess early or subclinical nephrotoxicity. Another important limitation is the inability to account for potential confounders such as concurrent nephrotoxic medications or individualized fluid management strategies, which could influence renal and electrolyte outcomes. Another limitation is that in a minority of cases, colistin was initiated empirically despite negative or inconclusive cultures, which may introduce selection bias.

Clinically, our findings support the cautious but justified use of colistin in neonates with MDR infections, provided that therapy is accompanied by intensive monitoring of renal function and electrolyte balance. Routine assessments of serum magnesium and sodium, in particular, should be integrated into treatment protocols, especially in very low‐birth‐weight infants.

Future prospective studies, ideally multicentric and with standardized monitoring of renal biomarkers, neurologic outcomes, and fluid‐electrolyte status, are needed to establish definitive safety profiles for colistin in neonates. Furthermore, exploration into optimal dosing regimens and co‐intervention strategies may help to minimize potential toxicity while preserving therapeutic efficacy. Refining treatment protocols, implementing proactive monitoring, and exploring adjunctive strategies will be essential to optimize the safety of colistin in this vulnerable patient population.

## Author Contributions

Conceptualization: Baran Cengiz Arcagok and Akan Yaman. Methodology: Baran Cengiz Arcagok. Validation: Baran Cengiz Arcagok, Akan Yaman, Ibrahim Kandemir, Nazli Jalalzada and Asli Memisoglu. Investigation: Ibrahim Kandemir, Nazli Jalalzada, Turkay Rzayev. Data curation: Ibrahim Kandemir, Turkay Rzayev. Writing – original draft preparation: Baran Cengiz Arcagok Writing – review and editing: Baran Cengiz Arcagok, Akan Yaman, Hulya Selva Bilgen, Asli Memisoglu. Supervision: Asli Memisoglu, Hulya Selva Bilgen. Project administration: Asli Memisoglu.

## Ethics Statement

The study was conducted in accordance with the principles of the 2008 Declaration of Helsinki. Ethics committee approval was obtained (Dean of Faculty Clinical Research Ethics Committee, Date: 02.07.2021; Number: 09.2021.885).

## Consent

Informed consent was obtained from the parents of the patients involved in this study.

## Conflicts of Interest

The authors declare no conflicts of interest.

## Data Availability

The data that support the findings of this study are available on request from the corresponding author. The data are not publicly available due to privacy or ethical restrictions.
